# Anti-Obesity Effects of *Grateloupia elliptica*, a Red Seaweed, in Mice with High-Fat Diet-Induced Obesity via Suppression of Adipogenic Factors in White Adipose Tissue and Increased Thermogenic Factors in Brown Adipose Tissue

**DOI:** 10.3390/nu12020308

**Published:** 2020-01-24

**Authors:** Hyo-Geun Lee, Yu An Lu, Xining Li, Ji-Min Hyun, Hyun-Soo Kim, Jeong Jun Lee, Tae Hee Kim, Hye Min Kim, Min-Cheol Kang, You-Jin Jeon

**Affiliations:** 1 Department of Marine Life Science, Jeju National University, Jeju 63243, Korea; hyogeunlee92@gmail.com (H.-G.L.); annie.lu1213@gmail.com (Y.A.L.); chanchanaichigua537@gmail.com (X.L.); localman@unist.ac.kr (J.-M.H.); 2 Marine Biodiversity Institute of Korea, 75, Jangsan-ro 101-gil, Janghang-eup, Seocheon 33362, Korea; Gustn783@mabik.re.kr; 3 Naturetech Co., 29-8, Yongjeong-gil, chopyeong-myeon, Jincheon 27858, Korea; jjlee@naturetech.co.kr (J.J.L.); taeheek@naturetech.co.kr (T.H.K.); kimhm@naturetech.co.kr (H.M.K.); 4 Research Group of Food Processing, Korea Food Research Institute, 245, Nongsaengmyeong-ro, Iseo-myeon, Wanju 55365, Korea

**Keywords:** red seaweed, *Grateloupia elliptica*, anti-obesity, adipocyte, adipogenesis, thermogenesis

## Abstract

Obesity is a serious metabolic syndrome characterized by high levels of cholesterol, lipids in the blood, and intracellular fat accumulation in adipose tissues. It is known that the suppression of adipogenic protein expression is an effective approach for the treatment of obesity, and regulates fatty acid storage and transportation in adipose tissues. The 60% ethanol extract of *Grateloupia elliptica* (GEE), a red seaweed from Jeju Island in Korea, was shown to exert anti-adipogenic activity in 3T3-L1 cells and in mice with high-fat diet (HFD)-induced obesity. GEE inhibited intracellular lipid accumulation in 3T3-L1 cells, and significantly reduced expression of adipogenic proteins. In vivo experiments indicated a significant reduction in body weight, as well as white adipose tissue (WAT) weight, including fatty liver, serum triglycerides, total cholesterol, and leptin contents. The expression of the adipogenic proteins, SREBP-1 and PPAR-γ, was significantly decreased by GEE, and the expression of the metabolic regulator protein was increased in WAT. The potential of GEE was shown in WAT, with the downregulation of PPAR-γ and C/EBP-α mRNA; in contrast, in brown adipose tissue (BAT), the thermogenic proteins were increased. Collectively, these research findings suggest the potential of GEE as an effective candidate for the treatment of obesity-related issues via functional foods or pharmaceutical agents.

## 1. Introduction

Over the past four decades, the populations of overweight and obese individuals have increased steadily in several countries [1,2,3]. The global health observatory (GHO) estimated that the global rate of adult obesity has nearly tripled since 1975, and the prevalence of overweight and obese individuals has risen in developed and developing countries [4]. According to the World Health Organization (WHO), obesity increases the risk of developing diseases such as hypertension, colorectal neoplasia, cancer, and neurodegenerative disease [5,6,7,8,9,10]. Hence, obesity has been recognized as a disease state by the American Medical Association and other organizations [11,12,13], and has emerged as a global health problem. Obesity is defined as having a body mass index (BMI) of 30 or more, and patients with obesity typically have high levels of triglycerides in their fat tissues owing to excessive food intake and a lack of exercise. Prolonged obesity can develop into chronic obesity, which may induce detrimental metabolic diseases, such as cardiovascular disease, diabetes mellitus, and increases the risk of many types of cancer, and also a lot of extraintestinal types [14,15,16,17,18]. Thus, several new anti-obesity agents have been developed to treat obesity and its complications [19]. Among the synthetic anti-obesity agents, Orlistat is the most common. However, this synthetic anti-obesity agent has known side effects, including gastrointestinal disorders and various types of pain [20,21,22]. Owing to the harmful side effects of synthetic agents, many researchers have focused on the development of natural anti-obesity agents that do not induce any adverse effects in the human body. Natural products, such as polyphenols, alkaloids, terpenoids, organosulfur compound, phytosterols, and bioactive compounds obtained from land-based and marine resources, were shown to exhibit anti-obesity effects through the regulation of obesity-related risk factors [23,24,25,26,27,28]. Marine seaweeds are a potential rich source of bioactive metabolites, including polyphenols, polysaccharides, and peptides, that exhibit antioxidant, anticancer, antidiabetic, anti-inflammation, anti-ultraviolet, and anti-obesity activities [29,30,31,32,33,34,35,36,37]. Many researchers have reported that marine seaweed metabolites, such as alginates, fucoidans, and phlorotannins, have potential anti-obesity effects in in vivo animal studies [38]. Among the seaweeds, recently, red seaweeds were shown to have anti-obesity effects [25,26]. In previous studies, it was revealed that an ethanol extract prepared from red seaweed exerted anti-adipogenic activities in 3T3-L1 adipocytes [25,26,38,39]. Therefore, this study investigated the anti-obesity effects of *G. elliptica* 60% ethanol extract (GEE) in high-fat diet (HFD)-induced obese mice.

## 2. Materials and Methods

### 2.1. Reagents

Dulbecco’s modified Eagle’s medium (DMEM), sera (fetal bovine serum (FBS) and bovine serum (BS)), including penicillin-streptomycin (P/S) supplements, were acquired from Gibco (Grand Island, NY, USA). Cell Signaling Technology (Bedford, MA, USA) supplied the primary and secondary antibodies used in the study for western blotting. Anti-obesity related antibodies were purchased from Cell Signaling Technology (Bedford, MA, USA). Reagents for 3T3-L1 cell differentiation, including 3-isobutyl-1-methylxanthine (IBMX), dexamethasone, and insulin, were obtained from Millipore Sigma (St. Louis, MO, USA). The serum insulin level analysis kit was purchased from Crystal Chem Inc. (Elk Grove Village, IL, USA). The cholesterol and the serum triglyceride levels were evaluated by using a colorimetric assay kit obtained from Abcam (Cambridge, MA, USA). Serum leptin was determined by using a kit from Invitrogen (Grand Island, NY, USA).

### 2.2. G. elliptica Ethanol Extract (GEE)

*G. elliptica* was collected from Jeju Island in Korea. The collected sample was completely washed with running tap water to remove epiphytes and salt and stored at −20 °C. The frozen samples were lyophilized by a freeze drying machine. The dried *G. elliptica* was homogenized with a grinder before extraction. For the preparation of *G. elliptica* 60% ethanol extract (GEE), the *G. elliptica* powder was extracted in 60% ethanol solution for 20 h at 70 °C and filtered through Whatman filter paper #4 (20–25 μm). The filtrate was concentrated by using a rotary vacuum evaporator. The concentrated extract was stored in a −80 °C freezer. The frozen extract was freeze-dried and homogenized for use in subsequent experiments.

### 2.3. Cell Culture and Differentiation

The 3T3-L1 cell line used in this study was purchased from the Korean Cell Line Bank (KCLB, Seoul, Korea). The cells were cultured in DMEM supplemented with 10% BS and 1% penicillin (100 units/mL)/streptomycin (100 μg/mL). The cells were grown in controlled conditions: humidity, 37 °C, and 5% CO2). Cell differentiation was initiated after 48 h when the cells reached 100% confluency. A DMEM growth medium with 10% BS growth serum, 1% antibiotics and differentiation solution (dexamethasone (0.25 μM), IBMX (0.5 mM), and insulin (5 μg/mL) which was used to induce cell differentiation. Further differentiation was induced through the addition of insulin (5 μg/mL) to the growth medium after 48 h. The culture medium was replaced every 2 days. At 8 days after cell differentiation, the cells were used for experiments.

### 2.4. Cell Viability Assay

The cytotoxicity of GEE was assessed via the 3-(4,5-dimethylthiazol-2-yl)-2,5-diphenyltetrazolium (MTT) assay, as previously described by Kang [26]. The cells were seeded in 48-well plates and GEE was treated at a range of concentrations. After a 48 h incubation period, MTT solution (dissolved in distilled water, 2 mg/mL) was added and incubated for a further 3–4 h. Subsequently, the plates were centrifuged (800 G, 5 min) and the supernatant was removed to dissolve the formazan crystal formed in living cells. To calculate cell viability, the relative quantities of formazan crystals were measured at 540 nm by using a microplate reader.

### 2.5. Oil Red O Staining

The relative lipid content accumulated in the 3T3-L1 cells was evaluated via the Oil Red O (ORO) staining method described by Kang [25]. Cells were stained with ORO, which specifically stain the lipid droplets in differentiated adipocytes. The 3T3-L1 cells were cultured in 12-well plates and differentiated. Each well was treated with 25, 50, 100, or 200 μg/mL GEE four times during adipocyte differentiation, except for the control group wells. After differentiation, the cells were washed with PBS and fixed in 10% formaldehyde for 1 h, washed in 60% 2-propanol, and dried at 25 °C. After drying, the cells were stained with 0.6% ORO solution for 1 h. The staining step was followed by several washing steps in distilled water. The cells was were then dried and photographed by using a Lionheart™ FX Automated Microscope (BioTek Instruments, Inc., Winooski, VT, USA). To quantify the lipid content, ORO stain in the 3T3-L1 cells was eluted with 100% 2-propanol for 1 h in a shaking incubator. Subsequently, the relative ORO content was measured at a wavelength of 500 nm (Synergy™ HT Multi-Detection Microplate Reader, Bio-Tek, Winooski, VT, USA).

### 2.6. Western Blotting Analysis

The harvested cells were washed with PBS and lysed in lysis buffer (20 mM Tris, 2 mM Na3VO4, 5 mM ethylenediaminetetraacetic acid (EDTA), 100 mM NaF, 10 mM Na4P2O7, 1 mM phenylmethylsulfonyl fluoride (PMSF), 10 mg/mL Aprotinin, 10 mg/mL leupeptin, 1% NP-40). The cell lysates were centrifuged at 12,902 G for 20 min at 4 °C, and the protein content was measured by using the bicinchoninic acid (BCA) protein assay kit. After protein quantification, the protein levels were normalized to 30 μg/mL and the lysate was mixed with sample loading buffer containing dithiothreitol (DTT) and heated at 99 °C for 3 min before the samples were subjected to SDS gel electrophoresis. The resolved proteins were electrophoretically transferred onto a nitrocellulose membrane (300 mA/60 min). After the proteins were transferred, non-specific binding to the membrane was blocked by incubation in 5% of skim milk in 1× TBST for 2 h. Subsequently, the primary antibody (1:1000 dilution) was introduced and incubated overnight at 4 °C, and the secondary antibody was added to the washed nitrocellulose membrane. The target protein bands were detected by using a Fusion Solo system (Vilber Lourmat, Marne-la-Vallée, France), activated by the application of enhanced chemiluminescence (ECL) western blotting detection kit.

### 2.7. Animals

Fifty C57 BL/6J male mice were purchased from the Jung Ang Lab Animal Inc (Seoul, Korea). The use of mice (5–6 weeks old, body weight 18–22 g) was approved by the Laboratory Animal Center of the Jeju National University. The experimental mice were housed under with optimal conditions for food (chow diet, high-fat diet), water, temperature (20 °C–22 °C), and humidity (55%) in a controlled room under a 12:12 h light/dark cycle. The chow diet consisted of nitrogen-free extract (60.7%), proteins (15.2%), cellulose (4.1%), minerals ash (5.0%), moisture (12.1%), and lipids (2.9%) and it’s caloric intake measured at 2793 kcal. The high-fat diet consisted of Casein, 30 Mesh (800 kcal), L-Cystein (12 kcal), Corn Starch (291 kcal), Maltodextrin 10 (400 kcal), Sucrose (691 kcal), Soybean Oil (225 kcal), Lard (1598 kcal), Vitamin Mix S10026 (0 kcal), DiCalcium Phosphate (0 kcal), Calcium Carbonate (0 kcal), Potassium Citrate, 1 H2O (0 kcal), Vitamin Mix V10001 (0 kcal), Choline Bitarrate (0 kcal), federal food, drug, and cosmetic act (FD&C) Red Dye #40 (0 kcal), and it’s caloric intake measured 4507 kcal. After a 2-week acclimatization period, the animals were divided into five groups (n = 5 in each cage) and subjected to different treatments. Each group was then assigned a diet as follows: chow diet (CD), high-fat diet (HFD), GEE (125 mg/kg) + HFD group (L-GEE), GEE (250 mg/kg) + HFD group (H-GEE), and *Garcinia cambogia* extract (125 mg/kg) + HFD group (GCE). Each morning the GEE was administered orally for 7 weeks. The mice were sacrificed after 7 weeks and the liver and adipose tissues were collected for further experiments. All experiments were performed in accordance with the experimental animal guidelines of Jeju National University animal center and were approved by the animal care and use committee (IACUC) of Jeju National University (Protocol 2018-0048)

### 2.8. Realtime polymerase reaction (RT-PCR) Analysis of messenger RNA (mRNA) Expression of Adipogenic Genes

The adipogenic and thermogenic gene expression was analyzed via RT-PCR techniques. To collect the total mRNA, 30–40 mg mouse tissues and 1 mL Trizol reagent (Invitrogen, Carlsbad, CA, USA) were placed in tubes containing steel beads (diameter, 1 mm) and the tissues were homogenized by using a bead beater (taco^TM^ Prep bead beater) for five cycles (40 s/cycle). The homogenized mixtures were then centrifuged (25,482× *g*, 4 °C, 20 min). After centrifugation, the supernatant was collected and the mRNA was extracted from the supernatant by using phase separation and RNA precipitation. The isolated total mRNA was converted to complementary DNA (cDNA) by using a cDNA reverse transcription kit (Takara, Shiga, Japan). Subsequently, the Thermal Cycler Dice Real-Time System (Takara, Shiga, Japan) was used to perform the reaction. The cDNA amplification was conducted in optimal conditions (enzyme activation (95 °C for 10 s), followed by 40 cycles of denaturation (95 °C for 5 s) and extension (58 °C for 10 s)). The sequences of used primers were as follows: GAPDH: forward, 5’-TGTGTCCGTCGTGGATCTGA-3’, reverse, 5’-TTGCTGTTGAAGTCGCAGGAG-3’; PPAR: forward, 5’-GTCACGGAACACGTGCAGC-3’, reverse, 5’-ACTCAGAAGTGGGCGAGGAC-3’; C/EBP-α: forward, 5’-GACTTCAGCCCCCTACCTGGA-3’, reverse, 5’-GTAGTCGTCGGCGAAGAGGT-3’.

### 2.9. Serum Analysis

Blood samples were collected from the mouse heart by cardiac puncture into an EDTA-rinsed syringe. The blood was centrifuged to collect serum (12,902× *g*, 20 min, 4 °C). The commercial analysis kits were used to evaluate the content of serum triglyceride (TG), total cholesterol (TC), leptin, and insulin.

### 2.10. Histological Analysis

Histological analysis was performed by the dissection of the liver tissue and white adipose tissue. The dissected tissues were stored in a solution of 70% ethanol before fixation. A 10% formaldehyde solution was used to fix the organs, and the organs were embedded in paraffin wax. Subsequently, the paraffin blocks were cut into 3 μm slices. Hematoxylin and eosin (H&E) was used to stain the paraffin sections, which were subsequently dried on a hot plate at 40 °C. All paraffin sections were observed by using an optical microscope equipped with a Cool SNAP-Pro color digital camera (Olympus, Japan).

### 2.11. Statistical Analysis

All results were analyzed in triplicate and presented as the mean ± standard deviation (SD). The analysis of statistical significance was performed by using statistical package for the social science (SPSS) software. Further, Student’s *t*-test or one-way analysis of variance (ANOVA) with Duncan’s multiple range tests were implemented in the significance evaluation. *p*-values (* *p* < 0.05, ** *p* < 0.01, *** *p* < 0.001, and **** *p* < 0.0001 compared with the control group, ^#^ *p* < 0.05, ^##^ *p* < 0.01, ^###^ *p* < 0.001, and ^####^ *p* < 0.0001 compared with the control group) of <0.05 were considered significant.

## 3. Results

### 3.1. Effect of GEE on Adipocyte Differentiation and Lipid Accumulation in 3T3-L1 Cells

**Figure 1:**
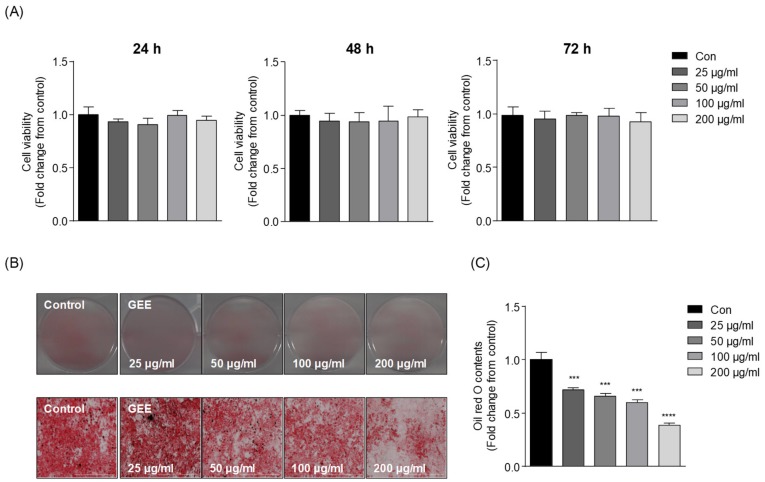
Effect of GEE (*G. elliptica* 60% ethanol extract) on intracellular lipid accumulation in 3T3-L1 cells: (**A**) The cytotoxicity of GEE on 3T3-L1 cells, (**B**) Microscopic images of stained 3T3-L1 cells, and (**C**) Quantification of lipid content. Data are expressed as the mean ± standard deviation (SD), (*n* = 3) in each group. Significant differences were identified at *** *p* < 0.001, and **** *p* < 0.0001, as compared to the control group. h, hour.

The cell viability was analyzed after treatment with GEE for 48 h. The viability of the GEE-treated cells relative to the control group is shown in Figure 1A. No concentration of GEE tested (25, 50, 100, or 200 μg/mL) exerted cytotoxic effects against 3T3-L1 cells. Thus, we selected all the treated concentrations for the subsequent experiments. The inhibitory effects of GEE on adipocyte differentiation and lipid accumulation were examined by using the ORO staining assay. As shown in Figure 1B, the microscopic image in the control group showed the high level of lipids stained. However, the treatments with GEE inhibited the lipid accumulations in 3T3-L1 cells. Furthermore, we quantified the lipid contents of 3T3-L1 cells. The relative intracellular lipid contents were shown in Figure 1C. In accordance with these results, GEE significantly reduced lipid accumulations by 28%, 34%, 40%, and 61% at the concentrations of 25, 50, 100, and 200 μg/mL, respectively.

### 3.2. In Vitro Expression of Adipogenic and Lipogenic Proteins after GEE Treatment

**Figure 2:**
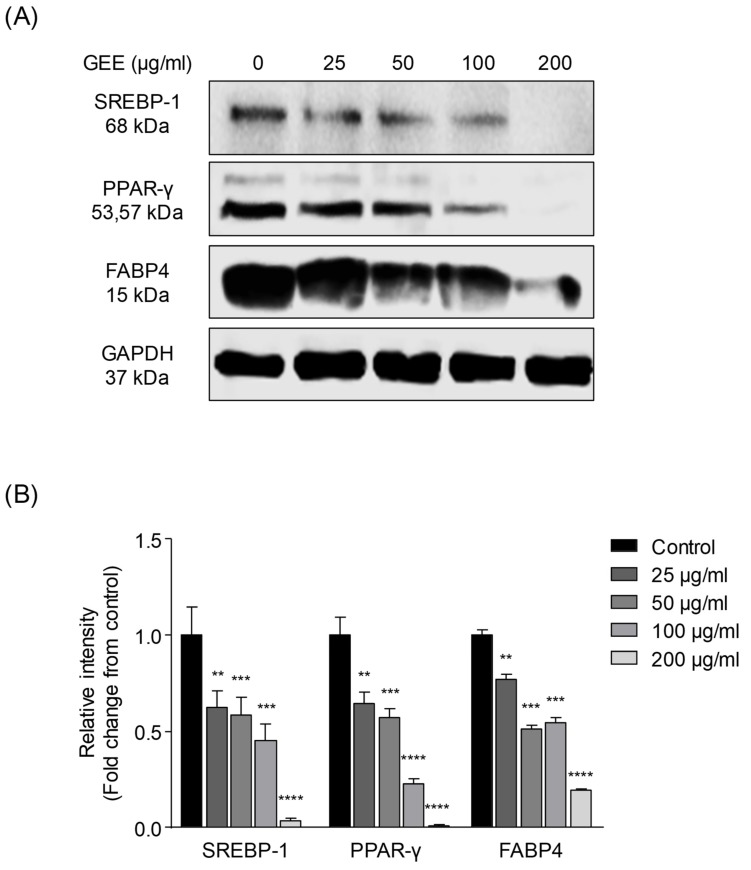
Effect of GEE on adipogenic protein expressions in 3T3-L1 cells: Western blot analyses of the sterol regulatory element binding protein-1 (SREBP-1), peroxisome proliferator activated receptor-gamma (PPAR-γ), fatty acid binding protein4 (FABP4) were performed and adipogenic protein bands were quantified and presented as graphs. (**A**) The western blot bands of adipogenic proteins, (**B**) quantification of lipid content. Data are expressed as the mean ± SD, (*n* = 3) in each group. Significant differences were identified at ** p < 0.01, *** p < 0.001, and **** p < 0.0001, as compared to the control group.

In order to determine whether GEE inhibits the expression of adipogenic proteins in differentiated adipocytes, western blotting was adopted. In this study, the adipogenic proteins including SREBP-1, PPAR-γ, and FABP-4 were examined. Zuo et al. and Shimano et al. reported that PPAR-γ was a key factor in adipocyte differentiation and SREBP-1 and FABP-4 had important roles in lipogenesis including in fatty acid synthesis, storage, and transportation in adipocytes [40,41,42,43,44,45,46,47]. The expression of adipogenic proteins was higher in the control group; however, the treatment with GEE significantly inhibited the expression of the adipogenic proteins, SREBP-1, PPAR-γ, and FABP-4, compared with that in the control group (Figure 2). As shown from the results, GEE has a potential to inhibit adipocyte differentiation as well as lipid accumulation in adipocytes. This activity was found to be mediated via the downregulation of adipogenic protein expressions.

### 3.3. Effects of GEE on Body and Adipose Tissue Weights in Mice with HFD-Induced Obesity

**Figure 3:**
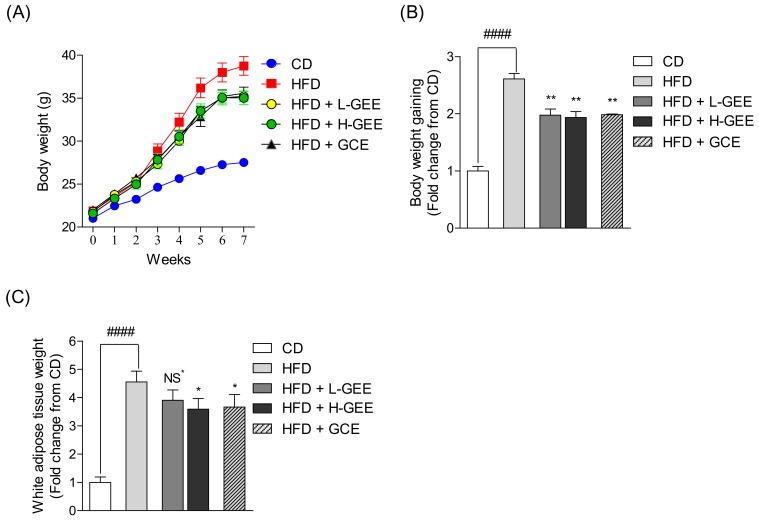
Effect of GEE on body weights and fat weight gain in high-fat diet (HFD)-induced mice: (**A**) Comparison of body weight, (**B**) body weight gain during 7 weeks, and (**C**) white adipose tissue weight at 7 weeks. Data are expressed as the mean ± SD, (*n* = 3) in each group. Significant differences were identified at * *p* < 0.05, ** *p* < 0.01, as compared to the HDF group, and ^#^ *p* < 0.05, ^####^ *p* < 0.0001, as compared to the control group. NS; Not Significant.

All the mice were orally administered with saline or GEE once per day for seven weeks and their body weights were recorded weekly. The body weights of mice in the HFD group were higher than in the CD group, which indicated the effect of the high-fat diet inducing obesity in C57 BL/6 mice after seven weeks. However, the body weights of mice in the GEE-treated groups to HFD mice were considerably reduced, compared with that in the HFD group (Figure 3A). Over seven weeks, the HFD group gained more body weight compared with the CD group; however, significant suppressions in the body weight gains were observed in the GEE-treated groups (Figure 3B). In addition, we investigated the weight of white adipose tissue in the mice with HFD-induced obesity. The WAT weight increase was remarkably reduced by H-GEE treatment and GCE as the positive control (Figure 3C).

### 3.4. Potential Action of GEE on White Adipose Tissue and the Adipogenic Proteins

**Figure 4:**
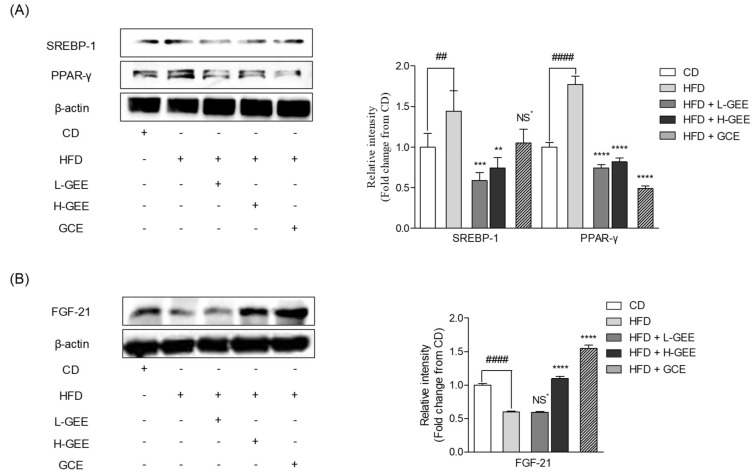
Effect of GEE on adipogenic SREBP-1 and PPAR-γ and metabolic fibroblast growth factor-21 (FGF-21) protein expression in mouse adipose tissue: (**A**) Adipogenic SREBP-1 and PPAR-γ expression, (**B**) metabolic FGF-21 protein expression in mouse white adipose tissue. Data are expressed as the mean ± SD, (*n* = 3) in each group. Significant differences were identified at ** *p* < 0.01, *** *p* < 0.001, and **** *p* < 0.0001, as compared to the HDF group, and * *p* < 0.05, ^#^ *p* < 0.05, ^##^ *p* < 0.01, ^###^ *p* < 0.001, and ^####^ *p* < 0.0001, as compared to the control group. NS; Not Significant.

To confirm whether GEE inhibits the expression of adipogenic proteins, the adipogenic tissues were analyzed by using western blotting techniques. As shown in Figure 4A, the expressions of SREBP-1 and PPAR-γ were higher in the HFD group than in the CD group. However, the oral administration of GEE significantly reduced SREBP-1 and PPAR-γ protein expression in white adipose tissues. These results demonstrated that GEE could regulate adipogenic proteins in mouse white adipose tissue.

### 3.5. Effect of GEE on the Expression of the Metabolic Regulator Protein FGF-21 in White Adipose Tissue

To investigate the effect of GEE on FGF-21, a metabolic regulator protein in white adipose tissues, we used western blotting analysis. Zhang and Li reported that FGF-21 was related to the lipid and glucose metabolism and energy homeostasis [48]. The protein expression of FGF-21 in the HFD group was lower than in the CD group (Figure 4B). However, the supplementation of H-GEE significantly increased FGF-21 protein expression in white adipose tissue. These results indicated that GEE had the potential to upregulate the FGF-21 protein expression in white adipose tissue. This may be the cause of the potential therapeutic effects on diabetes and obesity.

### 3.6. Inhibitory Effect of GEE on Expression of Adipogenic Genes in White Adipose Tissue

**Figure 5:**
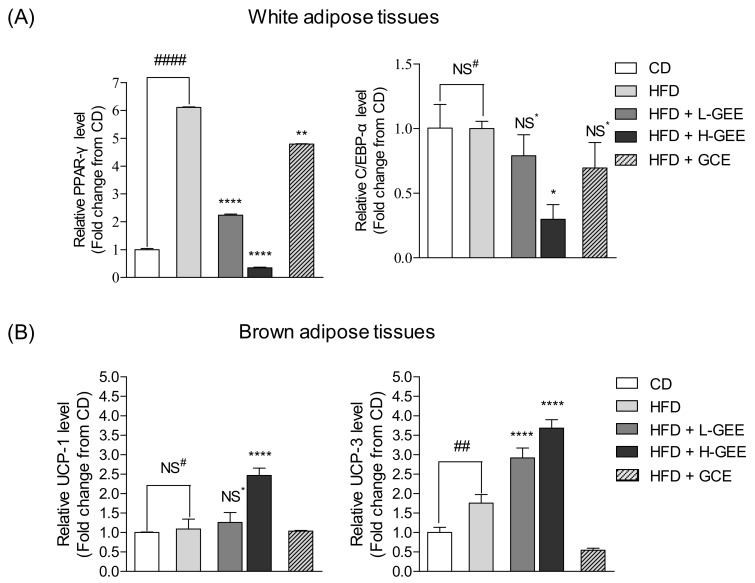
Effect of GEE on adipogenic PPAR-γ and C/EBP-α in white adipose tissue and thermogenic uncoupling protein-1 (UCP-1) and uncoupling protein-3 (UCP-3) mRNA expression in brown adipose tissues: (**A**) The mRNA expression level of PPAR-γ and C/EBP-α in white adipose tissue, (**B**) the mRNA expression level of UCP-1 and UCP-3 in brown adipose tissue. Data are expressed as the mean ± SD, (*n* = 3) in each group. Significant differences were identified at * *p* < 0.05, ** *p* < 0.01, *** *p* < 0.001, and **** *p* < 0.0001, as compared to the HFD group, and ^#^ *p* < 0.05, ^##^ *p* < 0.01, and ^####^ *p* < 0.0001, as compared to the control group. NS; Not Significant.

The analysis of cDNA by RT-qPCR was used to determine the expression of adipogenic genes. As shown in Figure 5A, PPAR-γ expression was significantly higher in the HFD group than in the control group and C/EBP-α protein expression was not changed by HFD-induced obesity. However, the supplementation of GEE significantly reduced PPAR-γ and C/EBP-α gene expression in white adipose tissue. These results indicated that GEE effectively reduced adipogenesis by the downregulation of the expression of the adipogenic genes, PPAR-γ and C/EBP-α, in HFD-fed obese mice.

### 3.7. Effect of GEE on Thermogenic Gene Expression in Brown Adipose Tissue

To investigate the effect of GEE on the expression of the thermogenic genes UCP-1 and UCP-3 in brown adipose tissue, total mRNA was extracted from mouse brown adipose tissue and cDNA was synthesized. cDNA was analyzed by RT-qPCR to determine thermogenic gene expression. As shown in Figure 5B, GEE activated the expression of UCP-1 and UCP-3, which are associated with energy expenditure through increased heat emission. In particular, H-GEE effectively increased UCP-1 and UCP-3 expression. These results indicated that GEE increased energy expenditure through the upregulation of the expression of thermogenic genes, including UCP-1 and UCP-3, in mice with HFD-induced obesity.

### 3.8. Effect of GEE on Mouse Blood Serum Biochemical Indices

The serum biochemical parameters were analyzed to confirm the anti-obesity effect of GEE (Table 1). The content of TG, TC, leptin, and insulin were successfully increased in the HFD-diet-fed group compared with those in the CD group. However, GEE administration significantly reduced TG, TC, and leptin content in the GEE-treated group compared with those in the HFD group. These results suggested that GEE treatment may have improved the serum TG, TC, and leptin levels in mice with HFD-induced obesity.

### 3.9. Histological Assay of Mouse Liver Tissue and White Adipose Tissue

**Figure 6:**
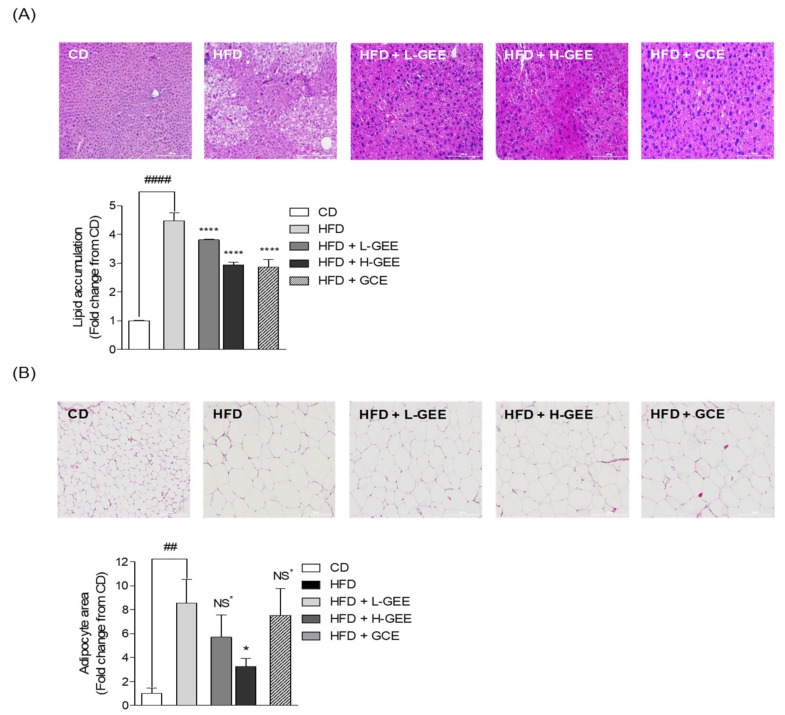
Histologic analyses of white adipose tissue and liver tissue in HFD-induced mice: lipid accumulation was expressed by measuring the area of lipid droplet in liver tissues and the size of white adipocyte was measured through imageJ software. (**A**) Histologic analysis of liver tissues, (**B**) white adipose tissues. Data are expressed as the mean ± SD, (*n* = 3) in each group. Significant differences were identified at * *p* < 0.05, ** *p* < 0.01, *** *p* < 0.001, and **** *p* < 0.0001, as compared to the HFD group, and ^#^ *p* < 0.05, ^##^ *p* < 0.01, ^###^ *p* < 0.001, and ^####^ *p* < 0.0001, as compared to the control group. NS, Not Significant.

The histological analyses of mouse liver and white adipose tissues were conducted using H&E staining. The histologic images revealed that the liver and white adipose tissue sections were stained with H&E (Figure 6). The results of the histological analysis in the liver tissue indicated that the HFD group showed increased fatty liver compared with the CD group. This demonstrated that lipid accumulation led to fatty liver, which was shown to cause hepatic steatosis in mice with HFD-induced obesity [49,50,51]. However, the treatments with GEE considerably reduced the lipid accumulation compared with that observed in the HFD group (Figure 6A). The degree of lipid accumulation in the white adipose tissue was reported to be proportional to the size of the tissue. Several researches have published this, with appropriate reference to the histological assays [52,53,54,55,56]. Therefore, we measured the size of the white adipocyte tissues by using ImageJ software. The size of the white adipocytes was higher in the HFD group than in the CD group, although the H-GEE group indicated a significant smaller size in adipocyte measurements compared to the HFD group (Figure 6B). These results indicated that GEE could regulate HFD-induced lipid accumulation in the liver and white adipose tissues and it could be reduced the risk of excessive adipocyte lipid accumulation and fatty liver diseases which could cause severe hepatic steatosis.

## 4. Discussion

Many researchers are interested in the prevention of obesity owing to the increased prevalence of obesity and obesity-related diseases [57]. Being obese or overweight can cause various diseases, including diabetes mellitus, gallbladder disease, cardiovascular diseases, and osteoarthritis in the human body [14,58,59]. Obesity has emerged as an important public health problem and there is a need for new strategies that provide effective solutions [60,61]. Obesity is described as diseases associated with excessive fat accumulation in adipose tissues and high levels of lipid and cholesterol in the blood. Excessive fat accumulation causes obesity-associated metabolic diseases, including blood lipid disorders, insulin resistance, and cardiovascular diseases. The cause of obesity cannot be defined by a single factor; indeed, it is well known that several factors are involved. However, the development of obesity is greatly influenced by dietary fat consumption. Several researchers have reported the harmful side effects of synthetic anti-obesity agents. Therefore, most research on anti-obesity has focused on the development of natural anti-obesity agents that exhibit safe anti-obesities without adverse effects. On the other hand, many studies have reported on the anti-obesity agents derived from land-based resources [40,41,42,43,44,45]; however, there are few reports from marine resources. This study targeted an anti-obesity agent from marine natural products. For the development of a marine-derived anti-obesity agent, the 60% ethanol extract of *Grateloupia elliptica* (GEE) was assessed in vitro and in vivo experiments.

*Grateloupia elliptica* (*G. elliptica*) is a red seaweed distributed in Korea and Japan. Particularly, a large production of *G. elliptica* was naturally produced in Jeju Island, S. Korea. It has been reported that *G. elliptica* has various biological activities such as anticancer, anti-diabetic, anti-inflammatory, antioxidant activities [46,47,48,49]. However, the lipid inhibitory effect of *G. elliptica* has not yet been fully investigated. Therefore, the potential anti-obesity effect of *G. elliptica* using 3T3-L1 preadipocyte and the high-fat-diet-fed obesity mouse model was investigated.

Fat accumulation is associated with the expressions of adipogenic proteins, which function as key regulators of cholesterol, lipid, and energy metabolism in the human body. Previous studies have revealed that adipogenic proteins including SREBP-1, PPAR-γ, and FABP4, were related to adipogenesis. Accordingly, most of the obesity research has focused on the inhibition of adipogenesis by suppression of those adipogenic protein expressions [50,51,52,53,54,55]. Some publications have found that the PPAR-γ activity was controlled by SREBP-1 affecting adipogenesis via the regulation of other adipogenic protein expressions [56,57] and the major adipogenic proteins such as PPAR-γ and C/EBP-α were synergistically activated [58,59]. Furthermore, the fatty acid binding protein 4 (FABP4), which transports fatty acids from the extracellular matrix to the intracellular matrix of adipocyte, is highly activated by PPAR-γ during the adipocyte differentiation [60,61]. Therefore, the regulation of adipogenic protein expression may be an effective strategy to control excessive lipid accumulation in adipocytes.

In this study, no cytotoxic effect was exhibited by GEE on 3T3-L1 adipocyte cells and the effect of GEE was predominantly observed on the intracellular lipid accumulation. In addition, GEE significantly decreased the expression of the adipogenic proteins SREBP-1, PPAR-γ, and FABP4 in 3T3-L1 cells. In vivo animal studies also revealed that the oral administrations of GEE at the two doses (the high dose, H-GEE and low dose, L-GEE) reduced HFD-induced body weights, body weight gains, fat weights, and serum contents of TG, TC, and leptin compared with that in the HFD group. Moreover, the GEE administrations effectively decreased adipogenic SREBP-1 and PPAR-γ protein expression, but increased the metabolic regulator protein, FGF-21, in white adipose tissue. Furthermore, adipogenesis and thermogenesis were further verified via the RT-qPCR analysis, including adipogenic PPAR-γ and C/EBP-α as well as the thermogenic UCP-1 and -3. The histological analyses indicated that GEE reduced the lipid accumulations in adipose tissues and liver tissues.

Taken together, our findings indicated that GEE significantly reduced adipogenic SREBP-1, PPAR-γ, and FABP4 protein expression in differentiated 3T3-L1 cells. Therefore, it has been observed that GEE effectively reduced the expressions of the adipogenic proteins both in vitro and in vivo. These adipogenic proteins influence accumulating fats in the body. Therefore, GEE inhibits the accumulation of fats via downregulations of those adipogenic proteins. Besides, UCP-1 and -3 as well as FGF-21 were activated by GEE, which means the energy consumption is increased. In conclusion, GEE could influence the fat accumulation and the energy expenditure via the fat metabolism. Collectively, these results indicate that GEE could regulate adipogenesis in 3T3-L1 cells and mice with HFD-induced obesity.

## 5. Conclusions

In conclusion, our findings demonstrated that ethanol extract of G.elliptica has inhibitory effect on lipid accumulation and adipogenesis in adipocyte and HFD induced obesity mice. These results suggest that the ethanol extract of G.elliptica could be used as a useful candidate for natural anti-obesity agents and functional foods to treat obesity.

## Figures and Tables

**Table 1:** Effect of GEE on mouse blood serum biochemistry in HFD-induced obese mice.

Parameters	Groups				
	CD	HFD	HFD + L-GEE	HFD + H-GEE	HFD + GCE
Triglyceride (mmol/μL)	65.51 ± 2.03	110.78 ± 1.62 ^####^	77.41 ± 0.14 **	58.22 ± 0.14 ***	94.43 ± 0.14 **
Total cholesterol (μg/μL)	38.75 ± 0.00	50.17 ± 0.05 ^##^	89.53 ± 0.17 **	29.33 ± 0.07 ***	53.66 ± 0.08 ^NS^*
Leptin (pg/mL)	212.50 ± 2.50	4308.13 ± 59.37 ^####^	3176.88 ± 44.38 ***	1896.88 ± 1.87 ***	4561.88 ± 4.38 **
Insulin (ng/mL)	1.48 ± 0.02	4.50 ± 0.18 ^####^	4.10 ± 0.05 ^NS^*	3.97 ± 0.00 ^NS^*	6.23 ± 0.18 **

Data are expressed as the mean ± SD, *n* = 3 in each group. Significant differences from the HFD group were identified at * *p* < 0.05, ** *p* < 0.01, and *** *p* < 0.001, as compared to the HFD group, and ^##^ *p* < 0.01, and ^####^ *p* < 0.0001, as compared to the control group. NS, Not Significant.

## Abbreviations

MTT	3-(4,5-dimethylthiazol-2-yl)-2,5-diphenyltetrazolium bromide
BS	bovine serum
CD	chow diet
DMSO	dimethyl sulfoxide
DMEM	Dulbecco’s modified Eagle’s medium
FABP4	fatty acid-binding protein 4
FBS	fetal bovine serum
GCE	garcinia cambogia extract
GAPDH	glyceraldehyde 3-phosphate dehydrogenase
GEE	Grateloupia elliptica ethanol extract
H&E	hematoxylin and eosin
HFD	high-fat diet
ORO	oil red o
PPAR-γ	peroxisome proliferator-activated receptor gamma
SREBP-1	sterol regulatory element-binding protein 1
TC	total cholesterol
TG	triglyceride
